# Event and Comment

**Published:** 1907-01-15

**Authors:** 


### DENTAL REGISTER.

Vol. LXI. January 15, 1907. No. 1.

### EVENT AND COMMENT.

### Institute of Dental Pedagogics.

The Fourteenth Annual Meeting of this organization has just been held in Chicago, with an attendance of over one hundred persons; coming from New Orleans, Boston, New York, Philadelphia and all the middle section of the country.

It is surprising what a live interest this organization has been able to keep up during all these years. It was predicted soon after its organization that it would work itself out in a few years; but if the last meeting is a criterion, it seems to be just getting fairly under way. The subjects that were up for discussion were old enough to be trite and uninteresting: but the objects aimed at were of such vital interest to those who took up the discussion that they appeared never to have been worked over before. One essayist dared to intimate that his topic had been discussed so much that, like the prophet of old, he only remained to champion a lost cause. Before the subject had been passed he was made to see that it was one of the livest subjects on the program, for it took an entire session and a banquet to stop the discussion. The one reason for the continued interest this organization maintains is that there seems to be a deep interest of everyone who attends these meetings in every topic that is brought up for discussion; whether it be the one he is engaged in teaching or not. The method advocated by one teacher for impressing his subject supon his pupils will have its lesson for every other teacher because it is the method that is important and not the subject.

For this reason the attendance was constant and the interest manifest all the time. It would seem that pedagogic principles ought to have a larger place in the considerations of such a convention; but it was clearly evident that individual methods that had produced results were more sought and created the greatest interest. This method of arriving at plans of teaching may not be so scientific, but they certainly lead up to the ultimate adoption of principles through personal demonstrations which have a more attractive influence for the average dental teacher than the pedantic methods of pedagogy.

Another important element in the success of this organization is the cordial and generous manner in which each contributor gives up his ideas when better ones are brought forward, it is true there are many warm contentions, but the idea of giving is so prevalent that selfishness and bigotry are practically submerged, and there prevails a spirit of good fellowship that if it were possible to transplant to the Faculties Association would mean tremendous advances in dental education in the near future. Why can't the Faculties Association repeal its laws and stop legislating and try to solve its problems on a pedagogic basis? The so-called rules and laws of the Faculties Association have made lots of trouble and seem destined to make more, and very little of permanent benefit can be charged up to the credit of educational advance because of its existance.

The fact is, that body was not spiritually conceived and it seems destined to die, because of its pagan tendencies, without accomplishing anything worth while. We predict a longer and more useful life for the Institute of Pedagogics which can't die so long as it maintains its present ideals and practices.

**Dental Text Books.**

The President of the Institute of Dental Pedagogics, in his annual address at the recent session of that body called attention to the fact that there was scarcely an acceptable text, or class-room book on any of our technical subjects, and in the discussion which followed there appeared less harmony of opinion than was indicated in the address. Some were too bulky and comprehend too many subjects, others were unscientific, and most of them were antiquated in in time or method. No remedy was apparent after an extended consideration and the matter was finally referred to a committee to consider and report at the next meeting. How this committee shall arrive at any satisfactory result remains for the future; but it would be a good plan to have everyone who thinks he knows what kind of a book he wants write out his ideas and hand them to this committee for suggestions on which to work.

The great criticism that can be made of our text books, so-called, is that they are made for two different purposes and people; to instruct the pupil in one case and to fortify the practitioner in the other. When both missions are attempted in a single book it is unintelligible to one and tiresome to the other. What we need is class books on the principles of our art for the pupils, and a treatise, on encyclopedic treatment with frequent revisions for the practitioners. Class books ought to be brief and exact in statement with marginal references to more exhaustive statements so as to prevent memorizing and to establish the habit of research early in the students career.

A new and better edition of the American System of Dentistry might meet the practitioners' needs: but a suitable class manual on any technical subject has yet to be made. It is a large task and every one shrinks from undertaking it, but it can better be done now than at almost any later time.

### Prohibition State Board Examinations.

We recently had submitted to us a set of questions set for the examination of candidates desiring to register for practice in a state whose law stated that the board's examination should consist of such questions as would test the applicants knowledge of the elemental principles for the practice of dentistry. A complaint had been made that the questions set by the board were such of a technical character as to practically exclude a large number of well qualified applicants from registration. As they could not answer correctly a sufficient number of the questions to pass the examination.

The questions referred to were pertinent and covered the subjects in a very satisfactory manner. There were no catch questions, and few that any well prepared recently graduated student should not answer without trouble. Some of the questions on chemistry and physiology, especially might be difficult for a practitioner, who had been out of college for a time

On the whole we considered them a very fair set of test questions and with an intelligent and considerate personal application they should not have been thought prohibitive.

We believe that our state boards need to get together in a kind of pedagogic session and learn to get at their work in a more satisfactory manner than is now the usual custom.

The candidates circumstances are an important consideration, but one man's circumstances are not to be considered when hundreds or thousands of human lives are an issue. No man should be licensed to practice dentistry who is a fool, a drunkard, or a scoundrel even if he is able to pass the examination. On the other hand some allowance should be made for the honorable and faithful practitioner who may not be able to give the anatomy of the medulla or enumerate the digestive ferments. It is high time our boards were taking into account the candidates power for good or evil as well as their culture and skill.

